# Multidrug resistant tuberculosis in Ethiopian settings and its association with previous history of anti-tuberculosis treatment: a systematic review and meta-analysis

**DOI:** 10.1186/s12879-017-2323-y

**Published:** 2017-03-20

**Authors:** Setegn Eshetie, Mucheye Gizachew, Mulat Dagnew, Gemechu Kumera, Haile Woldie, Fekadu Ambaw, Belay Tessema, Feleke Moges

**Affiliations:** 1 Department of Medical Microbiology, School of Biomedical and Laboratory Sciences, College of Medicine and Health Sciences, University of Gondar, Gondar Northwest, Ethiopia; 2 Department of Human Nutrition, College of Health Sciences, Debre Markos University, Debre Markos, Ethiopia; 3 Department of Human Nutrition, Institute of Public Health, College of Medicine and Health Sciences, University of Gondar, Gondar, Ethiopia; 4 Department of Nursing, College of Medicine and Health Sciences, University of Gondar, Gondar, Ethiopia; 5 WHO/TDR Clinical Research and Development Fellow at FIND, Geneva, Switzerland

**Keywords:** Multidrug resistant tuberculosis, Meta-analysis, Systematic review, Ethiopia

## Abstract

**Background:**

Efforts to control the global burden of tuberculosis (TB) have been jeopardized by the rapid evolution of multi-drug resistant *Mycobacterium tuberculosis* (MTB), which is resistant to at least isoniazid and rifampicin. Previous studies have documented variable prevalences of multidrug-resistant tuberculosis (MDR-TB) and its risk factors in Ethiopia. Therefore, this meta-analysis is aimed, firstly, to determine the pooled prevalence of MDR-TB among newly diagnosed and previously treated TB cases, and secondly, to measure the association between MDR-TB and a history of previous anti-TB drugs treatment.

**Methods:**

PubMed, Embase and Google Scholar databases were searched. Studies that reported a prevalence of MDR-TB among new and previously treated TB patients were selected. Studies or surveys conducted at national or sub-national level, with reported MDR-TB prevalence or sufficient data to calculate prevalence were considered for the analysis. Two authors searched and reviewed the studies for eligibility and extracted the data in pre-defined forms. Forest plots of all prevalence estimates were performed and summary estimates were also calculated using random effects models. Associations between previous TB treatment and MDR-MTB infection were examined through subgroup analyses stratified by new and previously treated patients.

**Results:**

We identified 16 suitable studies and found an overall prevalence of MDR-TB among newly diagnosed and previously treated TB patients to be 2% (95% CI 1% - 2%) and 15% (95% CI 12% - 17%), respectively. The observed difference was statistically significant (*P* < 0.001) and there was an odds ratio of 8.1 (95% CI 7.5–8.7) for previously treated TB patients to develop a MDR-MTB infection compared to newly diagnosed cases. For the past 10 years (2006 to 2014) the overall MDR-TB prevalence showed a stable time trend.

**Conclusions:**

The burden of MDR-TB remains high in Ethiopian settings, especially in previously treated TB cases. Previous TB treatment was the most powerful predictor for MDR-MTB infection. Strict compliance with anti-TB regimens and improving case detection rate are the necessary steps to tackle the problem in Ethiopia.

## Background

Despite the recent progress of global control efforts, tuberculosis (TB) remains a major public health threat, worldwide [1]. According to the World Health Organization (WHO) 2015 report, TB is the main cause of morbidity among millions of people each year and ranks alongside HIV as a leading cause of death worldwide. There were an estimated 9.6 million new TB cases, and there were also 1.5 million TB deaths, nearly 60% and 26.7% of deaths were reported among men and HIV positive people, respectively. Moreover, the report has also revealed that 5% of TB cases were estimated to have had multidrug-resistant TB (MDR-TB); 3.3% and 20% of MDR-TB cases were among new and previously treated TB cases, respectively [2].

TB continues to be one of the major public health problems in developing countries and is particularly compounded by high burdens of MDR-TB. Approximately, 75% of TB infections occur in Africa, South-East Asia, and Western Pacific regions where HIV is fueling the epidemic [3]. HIV is contributing to large increases in the incidence of TB, notably in Sub-Saharan Africa [4]. In Sub–Saharan Africa, pooled estimate of any drug resistant-TB prevalence among the new cases was 12.6%, and among previously treated patients was 27.2% [5].

The overall burden of MDR-TB is exceptionally high in resource limited countries, where health resources, finances, and the skilled personnel required for diagnosis and management are limited, making containment and the prevention of further spread more difficult [6]. In Ethiopia, the low socioeconomic status of the population, high prevalence of infectious diseases, poor treatment outcomes, longer treatment time (about two years), higher treatment costs, and many more complications make MDR-TB a more complex disease than TB [7, 8]. Based on a WHO report, Ethiopia is ranked as 15th among 27 countries with high burden drug resistant TB with an estimated 5200 cases occurring each year [9, 10].

In Ethiopia, TB has been recognized as one of the main public health threats since half century ago. The national TB control program has adopted a standardized TB prevention and control program so called directly observed treatment, short course (DOTS). At national level, there is one TB program manager who oversees a team of 11 TB program officers who are responsible for each region and work closely with the regional health bureau to maximize appropriate implementation of national TB control and prevention strategies. Ethiopia remains one of the high TB and MDR-TB burden countries where TB remains a significant cause of morbidity and mortality [11, 12].

Although MDR-TB is a growing concern in resource limited countries like Ethiopia, it is largely under-reported, compromising control efforts. Information concerning the true extent of the problem of MDR-TB in the African Region is limited. Since, there are significant gaps in surveillance, and lack of standards for methodology, data sharing and coordination. The overall epidemiology of drug resistant TB is not well understood in Ethiopia [13–16]. Hence, understanding the burden of the most prevailing infections like MDR-TB is urgently required to guide public health interventions that are both specific and effective. Therefore, the aim of this meta-analysis was firstly, to determine the pooled prevalence of MDR-TB among newly diagnosed and previously treated cases in Ethiopian settings and, secondly, to examine the relationship between previous TB treatment and MDR-MTB infection by conducting a systematic review and meta-analysis.

## Methods

### Settings

Ethiopia is a highly populated country in Africa, with 101 million lives in its nine regions and two federal cities. The operation of health system has been decentralized to regional governments and district health offices. Each district has a primary hospital with multiple health centers, and every health center is administratively linked to five health posts. Besides, health posts have been structured with two health extension workers who provide a package of up to 16 basic services including TB prevention and treatment follow up. The tuberculosis case definitions and management is as per the WHO TB treatment guidelines [11, 12].

### Definitions

In this meta-analysis, we have used the following terms; MDR-TB described as resistance to at least the two powerful first line anti-TB drugs (isoniazid and rifampin). According to WHO, MDR-TB among new cases is defined as resistance to isoniazid and rifampin drugs in patients that have never been treated for TB, and MDR-TB among previously treated TB patients, on the other hand, is defined as resistance to isoniazid and rifampin drugs in patients that have been treated for TB [17].

### Study selection

A literature search of the PubMed, Embase and Google Scholar databases was conducted, and studies potentially relevant to our study were identified. The search was carried out by 2 authors (SE, MD) independently, by using the following keywords; (MDR-TB or (Drug AND Resistant AND Tuberculosis)) AND (Associated AND Risk-factors) AND (Ethiopia). Among the citations extracted, abstracts were reviewed in an attempt to retrieve the clinical studies on MDR-TB among newly and/or previously treated TB patients. Studies that were relevant by title and abstract were accessed in full text to determine those that provided sufficient information to be included in our meta-analysis. Finally, the references cited by each eligible study were examined to identify additional articles. This systematic review and meta-analysis was done in accordance with QUOROM (Quality of Reporting of Meta-Analysis) guidelines [18].

### Inclusion and exclusion criteria

We have included studies carried out in Ethiopian settings, reporting MDR-TB prevalence and have sufficient data to calculate prevalence of MDR-TB among newly and/or previously treated TB cases. Studies were also only eligible if they reported the burden of MDR-TB for the past 10 years and were conducted in Ethiopia (institutional based findings, national surveys). Studies conducted among presumptive MDR-TB cases were excluded from the analysis, to minimize bias of reporting erroneously increased prevalence of drug resistant TB. Presumptive MDR-TB can be defined as: smear positive previously treated patients who define as relapse, return after default, and failure; new smear positive pulmonary TB patients who sputum remains smear positive at month 2 or 3 of treatment; symptomatic close contacts of known MDR-TB patient, and new smear positive with Human Immunodeficiency Virus (HIV) infected patients. Only studies published in English were considered.

### Outcome of interest

The main outcome of interest was the prevalence of MDR-TB among newly and/or previously treated TB patients. The prevalence was calculated by dividing the number of MDR-TB cases by the total number of TB patients. The results have been stratified by geographic location, types of TB cases (new versus previously treated TB cases), type of sample (sputum versus other possible specimens), and methods of MDR detection (culture alone versus mixed, i.e. both culture and molecular based methods). Secondly, we have also calculated the odds of developing MDR-TB among previously treated TB cases, compared with new TB cases.

### Data extraction and quality assessment

The relevant data from each selected study has been extracted independently by two authors (SE, BT) and summarized into an Excel spreadsheet. Discrepancies were resolved through consensus and discussion with a third author (FM). For each selected study, the following parameters were extracted: first author and reference, year of publication, study region/area, year/s/ of the study period, study patients as pulmonary tuberculosis (PTB) or extra PTB cases, types of sample utilized for MDR-TB isolation, total number of TB patients, and the method employed for MDR-TB detection (culture, DST and molecular typing). Moreover, we have also extracted the proportion of MDR-TB among new, previously treated and both cases, accordingly. To depict the time trends of the burden of MDR-TB in the country, we used the index year as the year that the study has been conducted not the year of publication.

The included papers were assessed for quality using a checklist based on Newcastle-Ottawa quality assessment scale. The quality of the selected studies was examined independently by two authors (SE, MG) using a set of predefined criteria, namely: clear data on the research design and study population (PTB versus extra PTB cases), quality of the reported data (stratified data for both new and previously treated cases), and the appropriateness of the method used for MDR-TB detection among newly diagnosed and previously treated TB cases.

### Statistical analysis

Since the included studies were conducted with different study designs and populations, we used a pooled random effects analysis to calculate the combined prevalence and the 95% confidence intervals (CI’s), by using the approach of DerSimonian and Laird [19]. We used Freeman Tukey arcsine methodology to address stabilizing variances [20]. The standard approach of inverse variance method to calculate pooled estimates and standard errors does not perform well in meta-analysis of prevalence. For prevalence near 0 or 1 (i.e., for studies with small or large prevalence), the inverse variance method adds disproportionately large weight, variance becomes small, and the calculated CI may lie outside of the 0 to 1 range. The double arcsine methodology corrects both variances instability and CI’s [21].

We assessed the heterogeneity among reported prevalence by computing values for chi-square (Q), I^2^ and *p*-values [22]. I^2^ ≥ 50% was considered as statistically significant. Possible sources of variation were explored using subgroup analysis by stratifying studies through predetermined variables; study regions, used methods, types of patients (new versus previously treated TB cases), and study population (≥ 100 and <100). The effect of small studies in publication bias was explored by the Begg’s funnel plot [23]. For time trends, model coefficients were transformed to rates and plotted against the index year along with the observed prevalence rates. Additionally, we have calculated the odds of MDR-TB infection among previously treated TB cases compared with new TB cases. Statistical analysis was accomplished using Stata v11 software package (Stata Corporation, College Station, TX), and Comprehensive Meta Analysis (version 3.1). *P*-values <0.05 were considered as statistically significant.

## Results

### Study characteristics

**Fig. 1:**
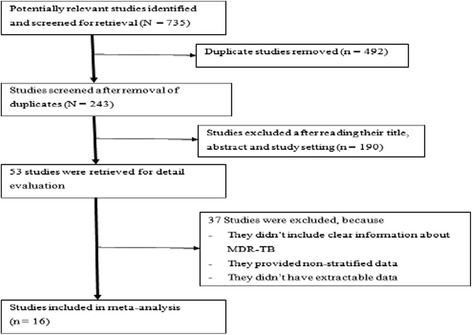
Flow chart shows selected articles for meta-analysis

Our electronic database search yielded 735 citations, of these 243 non-duplicate papers were assessed and 119 excluded after reviewing their title and study setting. The remaining 124 were examined by abstract screening of which 71 were excluded. The remaining 53 were initially considered as eligible by title and abstract for our meta-analysis, however after retrieving the full texts; only 16 studies met our inclusion criteria [8, 24–38] and were subjected to the meta-analysis. In brief, 15 of 53 studies were excluded because they did not include clear information regarding MDR-TB. 12 studies were excluded because they provided non-stratified data for the study participants, i.e. whether they were new or previously treated TB cases. Finally, 10 studies were not considered to have extractable data, though they did report the number or rate of MDR-TB cases (Fig. 1).

The included studies were published from 2008 to 2016 and reported data from 2002 to 2014. Of the 16 eligible studies, 12 reported MDR-TB data on both new and previously treated TB patients. Three studies have been done only among newly diagnosed TB cases, while 1 study has assessed drug resistant TB among previously treated TB cases (Table 1). The studies have provided MDR-TB data among a total of 151,065 TB patients. Of these, 12 studies reported among 150,395 newly diagnosed and previously treated TB cases. Most importantly, 3 studies exclusively reported data on 586 newly diagnosed TB cases, whereas 1 study was done among 84 previously treated TB cases. The majority of studies have assessed drug resistant TB in pulmonary TB cases; the remaining 1 study was done among extra pulmonary TB cases. Moreover, 14 out of selected studies, sputum was the most important sample for isolation of MDR-TB, while 1 study used fine needle aspirates, and type of sample was not specified for 1 study.

**Table 1:** Characteristics of included studies

References	Year of publication	Study area	Study population	Study design	Study period	Specimen	Methods employed	No of TB patients	New MDR-TB, %	Previously treated MDR-TB, %	Overall MDR-TB, %
Meskel et al. [25]	2008	Addis Ababa	Previously treated PTB patients	Cross-sectional study	2001 to 2002	Sputum	Microscopy, culture, and DST	84	-	26.2	26.2
WHO [12]	2008	Ethiopia (national survey)	Extra and PTB patients	Longitudinal cross-sectional study	2003 to 2006	N/S	Culture and DST	147,592	1.6	12	1.8
Yimer et al. [26]	2012	ARS major towns	New PTB patients	Cross-sectional study	2008	Sputum	Microscopy, culture, and DST	93	1.1	-	1.1
Tessema et al. [27]	2012	Gondar, Metema, Bahirdar & Debre Markos	PTB patients	Cross-sectional study	2009	Sputum	Microscopy, culture, genotype MTBC MTBDRs, DST	260	3.7	10.9	5
Abebe et al. [28]	2012	South western Ethiopia	New PTB patients	Cross-sectional study	2010 to 2011	Sputum	culture and DST	136	1.5	-	1.5
Hussein et al. [29]	2013	Bahirdar Fitche Ambo	PTB patients	Cross-sectional study	2011	Sputum	Microscopy, culture, RD9 typing and DST	102	11.8	11.1	11.8
Esmael et al. [30]	2014	Eastern ARS	PTB patients	Cross-sectional study	2010 to 2011	Sputum	Culture, and DST	230	1.8	18.5	6.5
Biadglegne et al. [31]	2014	Bahirdar, Gondar, & Dessie	TB lymphadenitis patients	Cross-sectional study	2012	Fine needle aspirates	Cytology, culture and GeneType MTBC assay &*Anti-TB drug resistant assay*	225	1.4	0	1.3
Seyoum et al. [32]	2014	Eastern Ethiopia	New PTB patients	Cross-sectional study	2011 to 2013	Sputum	Microscopy, culture and DST	357	1.1	-	1.1
Daniel et al. [33]	2014	Debre Birhan	PTB patients	Cross-sectional study	2013 to 2014	Sputum	Microscopy, culture, deletion and spoligotyping, DST	40	6.3	12.5	7.5
Tekle et al. [34]	2014	Benishangul-Gumuz & Awi	PTB patients	Cross-sectional study	2013 to 2014	Sputum	Microscopy, culture, RD9 typing and DST	87	1.3	8.3	2.3
Kebede [35]	2015	Ethiopia	PTB patients	Longitudinal cross-sectional study	2011	Sputum	Culture and DST	1422	2.7	17.9	5.1
Adane et al. [36]	2015	East Gojjam	PTB patients	Cross-sectional study	2011 to 2012	Sputum	Microscopy, culture, RD9 typing and DST	89	1.3	16.7	3.4
Maru et al. [37]	2015	Dessie	PTB patients	Cross-sectional study	2012 to 2013	Sputum	Culture, spoligotyping and DST	118	0	13.3	1.7
Mekonnen et al. [38]	2015	North Gondar	PTB patients	Cross-sectional study	2014	Sputum	Microscopy, Gene x-pert, culture and DST	124	2.3	13.9	5.6
Hamussie et al. [39]	2016	Arsi Zone	PTB patients	Cross-sectional study	2013 to 2014	Sputum	Culture and DST	106	2.4	14.3	4.7

Summary prevalence estimates are presented in Table 2, 14 studies have been conducted in different regions of Ethiopia; whereas two were national wide survey studies. The pooled prevalence of MDR-TB among 14 studies which were conducted in various regions of the country was 4.6% (95% CI 3.7% - 5.5%), compared with 1.9% (95% CI 1.8% - 2%) from studies conducted at national level. The observed difference was statistically different (*P* = 0.016). Interestingly, the prevalence of MDR-TB among studies having study participants 100 or above was 1.9% (95% CI 1.9% - 2%), this was significantly lower than a prevalence calculated from studies with their study participants less than 100 patients, 7.9% (95% CI 5.2% - 10.6%). Regarding the microbiological assays used in studies included in our meta-analysis, 8 studies have exclusively used culture to identify MDR-MTB, 8 studies used both culture and molecular methods (PCR based tests). Of note is that culture alone yielded a prevalence of 1.9% (95% CI 1.9% - 2%), which was statistically lower from the 4.3% (95% CI 3.1% -5.5%) of MDR-MTB detection using both culture and molecular methods.

**Table 2:** Summary estimates of included studies

Characteristics	Studies	TB patients	Combined effect (95%Cl)	*P*-value
Study population				
Studies ≥100 patients	11	150,672	1.9% (1.9% - 2%)	<0.001
Studies <100 patients	5	393	7.9% (5.2% -10.6%)	
Geographic region				
Studies from various regions	14	2051	4.6% (3.7% - 5.5%)	0.016
National level surveys	2	149,014	1.9% (1.8% - 2%)	
MDR-MTB isolation				
Culture only	8	150,020	1.9% (1.9% - 2%)	0.010
Mixed^a^	8	1045	4.3% (3.1% - 5.5%)	
Types of patients				
New cases	14	146,320	2% (1% - 2%)	<0.001
Previously treated cases	12	7584	15% (12% - 17%)	
All studies	16	151,065	6% (4% - 8%)	

Keys: ^a^Culture and molecular tests, *SE* standard error, *CI* confidence interval

**Fig. 2:**
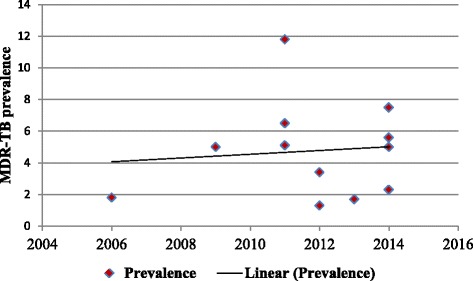
Time trends of MDR-TB prevalence: Observed and fitted estimates

Additionally, among the eligible studies, 12 were selected to determine the time trend of MDR-TB prevalence since the selected studies have data for MDR-TB rate in both newly and previously treated TB cases [8, 26, 28–30]. As indicated in Fig. 2, we used the index year of the studies, as described in the Methods section, and we observed no significant time-trend (*P* = 0.770).

### Prevalence of MDR-TB among newly diagnosed and previously treated TB cases

**Fig. 3:**
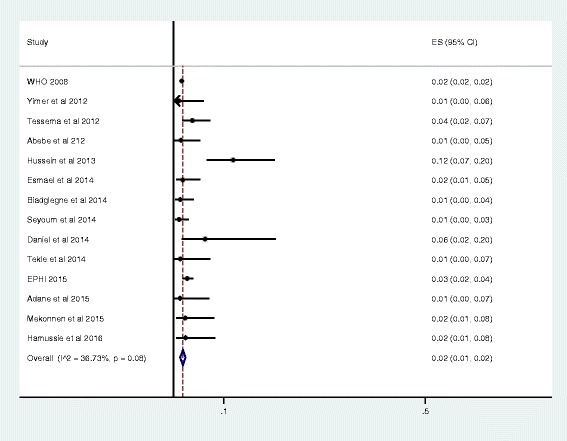
Forest plot of the pooled prevalence of MDR-TB among new TB cases

**Fig. 4:**
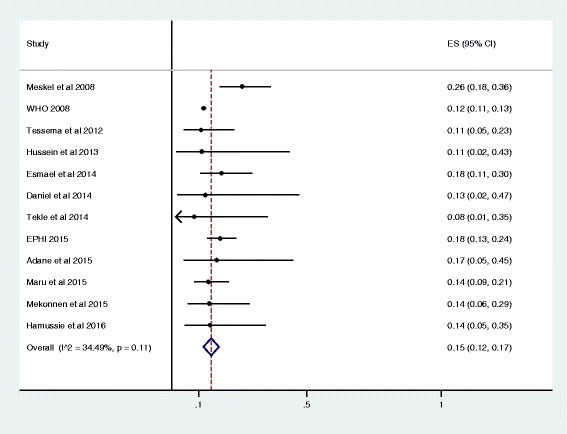
Forest plot of the pooled prevalence of MDR-TB among Previously treated TB cases

**Fig. 5:**
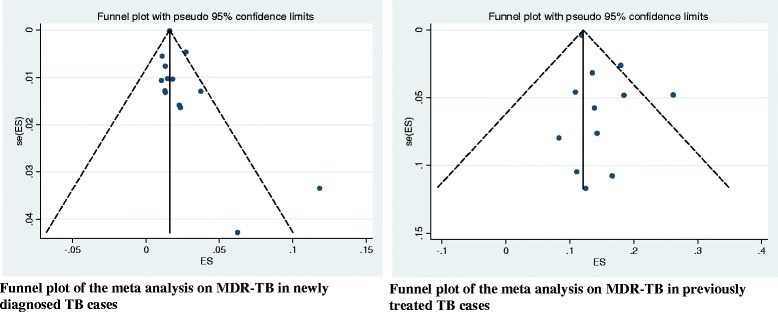
Funnel plots, exploring publication bias for the analysis of pooled estimate

As presented in the forest plot (Fig. 3), the pooled prevalence of MDR-TB among newly diagnosed TB cases was 2% (95% CI 1% - 2%). We assessed the heterogeneity among reported prevalence using the I^2^ statistic, and it was marginally insignificant (I^2^ = 36.7%, *P* = 0.08). Furthermore, we have also calculated the overall prevalence of MDR-TB among previously treated TB cases; it was 15% (95% CI 12% - 17%). The heterogeneity test indicated that no statistical significant variation among pooled estimate of MDR-TB in previously treated cases ((I^2^ = 34.5%, *P* = 0.11, Fig. 4). As shown in Table 2, the observed variation of MDR-TB prevalence among newly diagnosed and previously treated cases was statistically significant (*P* < 0.001). Moreover, we explored graphically the possibility of a publication bias. We did not observe an indication of such a bias in the studies included to calculate the pooled prevalence of MDR-TB among newly diagnosed and previously treated cases, accordingly (Fig. 5).

### Association between previous TB treatment and MDR-TB infection

**Fig. 6:**
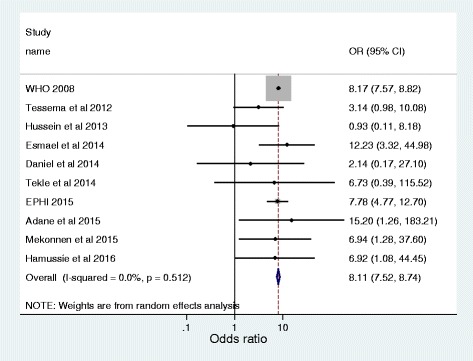
Forest plot of the pooled odds ratio indicating the association of previous TB treatment with MDR-MTB infection

**Fig. 7:**
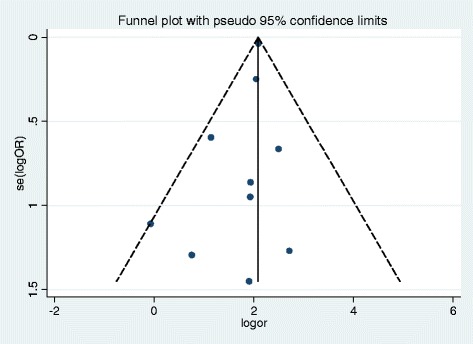
Funnel plot, exploring publication bias for the analysis of odds ratio

Across 10 studies with analyzable data that are summarized in Table 3 [8, 26, 28, 29, 32–35, 37, 38], we have assessed the burden of MDR-TB infection among previously treated cases, compared with new TB cases. Hence, we found that the odds ratio for MDR-TB infection among patients who were previously treated with anti-TB treatment was 8.1 (95% CI 7.5–8.7) compared with patients who were new cases. In clinical terms, this indicates that previously treated TB patients are 8.1 times more likely to develop a MDR-TB infection compared with newly diagnosed TB patients. Additionally, I^2^ statistic uncovered no variation among including studies (I^2^ = 0, *P* = 0.52, Fig. 6), and symmetry of funnel plot shows small study bias yielded insignificant effect (Fig. 7).

**Table 3:** Individual study data to calculate the odds ratio of MDR-MTB infection

S. No	Study name	Method	MDR-TB/previously treated TB cases	MDR-TB/newly diagnosed TB cases
1	WHO [12]	Culture	861/7271	4964/306990
2	Tessema et al. [27]	Culture & PCR based	5/46	8/214
3	Hussein et al. [29]	Culture & PCR based	1/9	11/93
4	Esmael et al. [30]	Culture	12/65	3/165
5	Daniel et al. [33]	Culture & PCR based	1/8	2/32
6	Tekle et al. [34]	Culture & PCR based	1/12	1/75
7	EPHI [35]	Culture	39/217	33/1205
8	Adane et al. [36]	Culture & PCR based	2/12	1/77
9	Mekonnen et al. [38]	Culture & PCR based	5/36	2/88
10	Hamussie et al. [39]	Culture	3/21	2/85

## Discussion

Multidrug resistant tuberculosis is an increasing global problem, with most cases are arising due to combinations of prescription errors and poor patient adherence. The epidemiology of MDR-TB varies significantly from country to country and region to region. As with all TB, 99% of MDR-TB occurs in high-burden resource-poor countries [14, 39]. Although, Ethiopia is one of the high TB/HIV and multidrug resistant TB (MDR TB) burden countries [13, 40], there is a still a large gap in knowledge about drug resistant TB in the country [14, 15]. So far only two nationwide surveillance studies were conducted to explore the overall epidemiology of MDR-TB in the country. However, in order to determine the current situation of MDR-TB in Ethiopia, up to date and evidence based studies are critically needed. Thus, we conducted this meta-analysis to estimate the burden of MDR-TB in newly diagnosed and previously treated patients in Ethiopian settings. Moreover, this study aimed to explore the significance of previous history of anti-TB treatment on MDR-MTB associated infections, and model its course over time.

Based on this meta-analysis, the pooled prevalence of MDR-TB among newly diagnosed and previously treated TB cases was 2% (95% CI 1% - 2%) and 15% (95% CI 12% - 17%), respectively. It is comparable with a previous meta analysis report that was documented in Sub-Saharan Africa countries, where HIV co-infection is rampant [5]. The report indicated that pooled estimate of MDR-TB prevalence among the new cases was 1.5% (95% CI 1.0–2.3), and among previously treated patients this was 10.3% (95% CI 5.8–17.4%). Likewise, anti-tuberculosis survey in Benin showed that the prevalence of MDR-TB among new and previously treated causes were 1.6% and 11.1%, respectively [41]. Similarly, national wide surveys have been also conducted in Mozambique and Rwanda, the prevalence of MDR-TB among new and previously treated cases were respectively 3.5% and 11.2%, and 3.9% and 9.4% [42, 43]. Drug resistant TB is unsurprisingly significantly associated with the HIV epidemic [44, 45]. There were an estimated 1.1 million HIV positive new TB cases globally in 2011. Around 79% of patients live in Sub-Saharan Africa [46]. It is understood that HIV infection causes suppression of immunological responses which subsequently leads to overwhelming infection by opportunistic and drug resistant strains [47]. According a 2008 WHO report, MDR-TB has been shown to be almost twice as common in TB patients living with HIV compared to TB patients without HIV [8]. Likewise, several studies demonstrated the association of drug-resistant TB with HIV infection [25, 48–51].

However, the pooled prevalence of MDR-TB among newly diagnosed and previously treated TB cases in the present study; was comparatively higher than that reports noted in economically developed countries [52–54]. With the availability of high standard diagnostic tests and effective treatment high income countries are moving towards MDR-TB elimination [15, 55]. Moreover, the range of available resources and specific TB control measures makes effective treatment of MDR-TB possible [15, 55]. Despite the introduction of direct observation of treatment (DOT) poor adherence remains a major obstacle to fight drug resistance TB in countries with poor socio economic circumstances like Ethiopia. The reasons for non-adherence are complex and involve more than the patients’ personal characteristics and attitudes. Studies have reported low literacy levels, household members’ fear of catching the disease, discriminatory behavior by health care providers, delays in care seeking behavior and self-denial due to stigma experienced by TB patients as some of the challenges facing adherence to TB treatment [15, 56, 57].

According to subgroup analysis, we observed that the pooled estimate of MDR-TB was significantly higher among studies with sample sizes of less than 100 participants, compared to studies where 100 or more participants were recruited. It is known that variation in sample size has a great effect on estimation. And possibly, the included studies have been carried out in different geographical locations that could have also impact on the observed prevalence. The calculated estimate of MDR-TB was also significantly higher in studies other than the two national surveys. Among the selected studies, 14 have been conducted either in hospital setups or other health institutions, and possibly results high estimate. Moreover, the prevalence of MDR-TB was higher among studies that have used both culture and molecular based detection methods compared to studies used culture alone. The significant variation could be presumably the fact that molecular based assays are the most powerful and highly sensitive techniques compared to conventional methods.

A second finding that highlights the association of previous history of anti-TB treatment with MDR-TB infection was high. As indicated in the study, the observed prevalence of MDR-TB was significantly higher than that among newly diagnosed TB cases (15% versus 2%, *P* < 0.001). Most importantly, TB patients who had history of anti-TB treatment exposure were 8.1 times more likely to develop MDR-TB infection compared with newly diagnosed TB cases. Previously treated patients often constitute a very heterogeneous group including those who experience relapse after receiving successful treatment, those who return after default, and those who start receiving a re-treatment regimen after having experienced previous treatment failure [58]. There are large evidences that noted that history of anti-TB exposure is one of the main contributing factors in acquisition of MDR-TB [9, 27, 59]. Multidrug resistance among previously treated TB patients usually results from exposure to a single drug that suppresses the growth of bacilli susceptible to that drug but permits the multiplication of pre-existing drug-resistant mutants [60]. It is the most common type of resistance to the first-line drugs and can emerge against any anti-tuberculosis agent during chemotherapy. The occurrence of MDR- TB is also attributable to lack of standard based drug prescription, probably leads to treatment failure and intensifies the population drug resistant strains [61].

Furthermore, we evaluated the time trend of MDR-TB prevalence from 2006 to 2014, which is shown to be stable, does not reflect possible changes in the implementation national TB control programs. A recent global report revealed that the Millennium Development Goal 6 targets on reducing the TB incidence rate has already been accomplished in Ethiopia [40]. Nationally, the TB incidence rate has fallen to 224 per 100,000 of the population in 2013 compared 369 in 1990 [40]. However, the issue of drug resistance in the country remains in question. The low level of reported drug resistance in Ethiopia may not be an accurate reflection of reality, and the limited evaluation may be responsible for this skewed portrayal. In 2010, less than 5% of new and previously treated TB patients were tested for MDR-TB because of limited availability of the test in most developing countries. Specifically, in Ethiopia the ratio of laboratories capable of performing MTB culture and capable of running line probe assay for detection of MDR-TB was 0.1 per 5 million populations. Besides, lack of research based national drug resistance survey data is a barrier to understanding the magnitude of prevalence and incidence of MDR [6, 14, 15].

## Limitation of the study

It should be noted again that a limitation of English literature was posed in our meta-analysis. Also, our analysis was limited by the quality of the included studies and by the fact that many of the DR-TB surveys identified during our searches were excluded because they had not stratified patients according to their treatment history. The existing literature could not be used to depict the exact geographic distribution of MDR-TB prevalence in newly diagnosed and previously treated TB patients, since we did not review data on some regions of the country (such as, Tigray, Afar, Somali, & Gambela). Also, there were some methodological differences between studies.

## Conclusion

Our analysis showed that the burden of MDR-TB remains a huge concern in Ethiopian settings. Particularly, the prevalence of MDR-TB was higher among previously treated TB patients. As detailed earlier, previously treated TB patients were 8.1 times more likely to develop a MDR-TB infection compared with newly diagnosed TB patients. Moreover, in the past 10 years, the time trend of MDR-TB prevalence was stable. Therefore, this finding underscores the importance of reducing the burden of MDR-MTB infection in Ethiopian settings. Effective implementation of DOTS program, strict compliance with infection control policies, and enhancing case detection rate through strengthening the TB laboratory capacity including introduction of rapid molecular diagnostic methods and implementation of active case finding through MDR-TB contact screening, seem to be the necessary next steps in this effort.

## Abbreviations

CI: Confidence interval
DOTS: Directly Observed Treatment, Short-course
DST: Drug susceptibility testing
HIV: Human immuno-deficiency virus
MDR: Multi drug resistant
MDR-MTB: Multidrug resistant *Mycobacterium tuberculosis*
MDR-TB: Multidrug resistant tuberculosis
MTB: *Mycobacterium tuberculosis*
PCR: Polymerase chain reaction
PTB: Pulmonary tuberculosis
TB: Tuberculosis
WHO: World Health Organization
